# Attitudes and practices of ophthalmology doctors in London (UK) on the importance of discussing work as a clinical outcome with patients during consultations: A cross sectional survey

**DOI:** 10.1371/journal.pone.0268997

**Published:** 2022-06-09

**Authors:** Katherine Kuncewicz, Isabelle Schiff, Jaroslaw Liszka, Sheni Akinfie, Nadia McLurcan, Ira Madan, Shairana Naleem, Vaughan Parsons

**Affiliations:** 1 GKT School of Medical Education, King’s College London, London, United Kingdom; 2 Occupational Health Service, Guy’s and St Thomas NHS Foundation Trust, St Thomas Hospital, London, United Kingdom; 3 Faculty of Life Sciences and Medicine, King’s College London, London, United Kingdom; University of Botswana Faculty of Medicine, BOTSWANA

## Abstract

**Background:**

Limited research suggests that non-occupational health doctors rarely discuss occupation with their patients. There is a gap in research regarding the attitudes and practices of doctors towards discussing patient occupation and return to work. The aim of this work was to explore the attitudes of ophthalmology doctors towards work as a clinical outcome and assess the need for occupational health training among participants (doctors).

**Methods:**

A cross-sectional survey among doctors working in ophthalmology in two London teaching hospitals. The survey focused on the attitudes of doctors towards ‘work’ as a clinical outcome, their practices of asking patients about occupation, their perceived level of competency in this area of clinical practice and the level of training doctors had received in this field. Descriptive data analysis was undertaken and results presented as frequencies and proportions.

**Results:**

The response rate was 30/72 (42%). Approximately a quarter (8/30;27%) of doctors ‘always’ discussed return to work during care planning whilst the majority (25/30;87%) of doctors agreed or strongly agreed that this should always be the case. Over half of the doctors had received no formal OH training on how to discuss or assess the impact of health on work and only 17/30 (57%) considered themselves competent in discussing these work outcomes with patients. Over half agreed that additional training would be useful, with the majority believing that it would be most useful at all stages of medical training.

**Conclusion:**

We found the majority of ophthalmology doctors regard ‘return to work’ as an important clinical outcome yet most do not routinely discuss work outcomes with patients to inform care planning. Majority of doctors lack training in how to discuss issues relating to work and would benefit from additional OH training.

## Introduction

The health and economic benefits of work are well established [1–3]. Research shows that being out of work is associated with poor physical health (such as increased blood pressure, serum cholesterol as well as increased risk of death due to cardiovascular disease) and mental health [4–7], and harmful behaviours including increased alcohol use and smoking [8]. Conversely, returning to work after sickness absence appears to have a positive effect on mental health [9]. Despite the protective benefits of work, limited research suggests that work-focused discussions do not routinely occur between patients and doctors in primary and secondary care [10, 11]. Clinical practice reforms in the UK regarding sickness certification for workers highlights the prominent role that doctors can play in advising what work activities patients are able to perform, particularly if reasonable work adjustments are made (e.g. phased return to work, workplace adaptions or amended duties), provide an opportunity for doctors to understand their patients’ job requirements and to discuss and agree on work expectations, and to also consider any psychological obstacles which may be impacting return to work. [12] This reform was driven by the recognition that ‘health’ and ‘work’ are intrinsically linked and emphasised the role that doctors can play in assessing the work ‘functional’ ability of patients. A key feature of this reform was the introduction of the fit note certification, replacing the traditional sick note certificates, which uses the term ‘may be fit’ for work to categorise a patient’s capacity to work [2]. However, since its introduction very little is known about the attitudes and work practices of doctors taking on this role. An audit of patient records from three General Practices in England found the recording of patient occupation to be “infrequent and inconsistent”, with the frequency of recording being between 1 to 30% [10].

The aim of this work was to explore the attitudes of ophthalmology doctors towards ‘work’ as a clinical outcome and to assess the need for occupational health training among participants (doctors) Our decision to focus on ophthalmology doctors was to allow comparisons to be made with previous studies exploring attitudes toward work as a clinical outcome and OH training among doctors from other specialties.

## Methods

A cross-sectional study design was used. We developed a 16-item questionnaire which was piloted with two clinicians (one each from Guy’s and St Thomas and King’s College London NHS Foundation Trusts) in order the gather feedback on this readability and to allow us to make very minor refinements before administering it using a convenience sampling frame. The questionnaire comprised of three areas of interest. These included basic work-related demographics, perceptions of the importance and frequency of doctor-patients discussions regarding work as a clinical outcome, and provision of OH training to enhance clinical practice in Ophthalmology. Ophthalmology doctors employed at two large teaching hospitals in London (UK) were invited to participate using a convenience and snow-ball sampling frame ie. we sent via email an electronic copy of the participant information sheet which included a link to the online survey to the two clinical leads at each institution with a request that they promulgate this information via email among their clinical teams. Paper copies of the questionnaire were also made available. Several email reminders were also used to optimise the response rate as this was shown to be effective in an early study [13].

Descriptive statistical data analysis was used, with results presented as proportions of overall responses. Data was analysed in percentages on Microsoft Excel. There was no missing data from the cohort who responded, as all subjects completed all questions. We analysed only based on those who responded. No personal identifiable information (including age) was collected. An online questionnaire and information sheet were emailed with paper forms made available, with several reminders sent to encourage participation. The questionnaire was open between 11–27 November 2020.

Ethics statement: This project met the criteria of a service evaluation as defined by the National Health Service (NHS) Health Research Authority in the UK so did not require ethics review/approval to conduct. Accordingly, this project was registered as a service evaluation project (ref: 11388) with the clinical audit team at Guy’s and St Thomas NHS Foundation Trust. Completion of the questionnaire following provision of the project’s information sheet was taken as consent to contribute individual data.

## Results

There were 20/51 doctors at Guy’s and St Thomas’ NHS Foundation Trust and 10/21 doctors at King’s College Hospital who completed the survey, giving an overall response of 42% (39% and 48% respectively), which is considered accepted for online surveys [14]. There are only around 1,260 ophthalmologists in the entirety of the UK, therefore it is a reasonable sample size [15].

**Fig 1:**
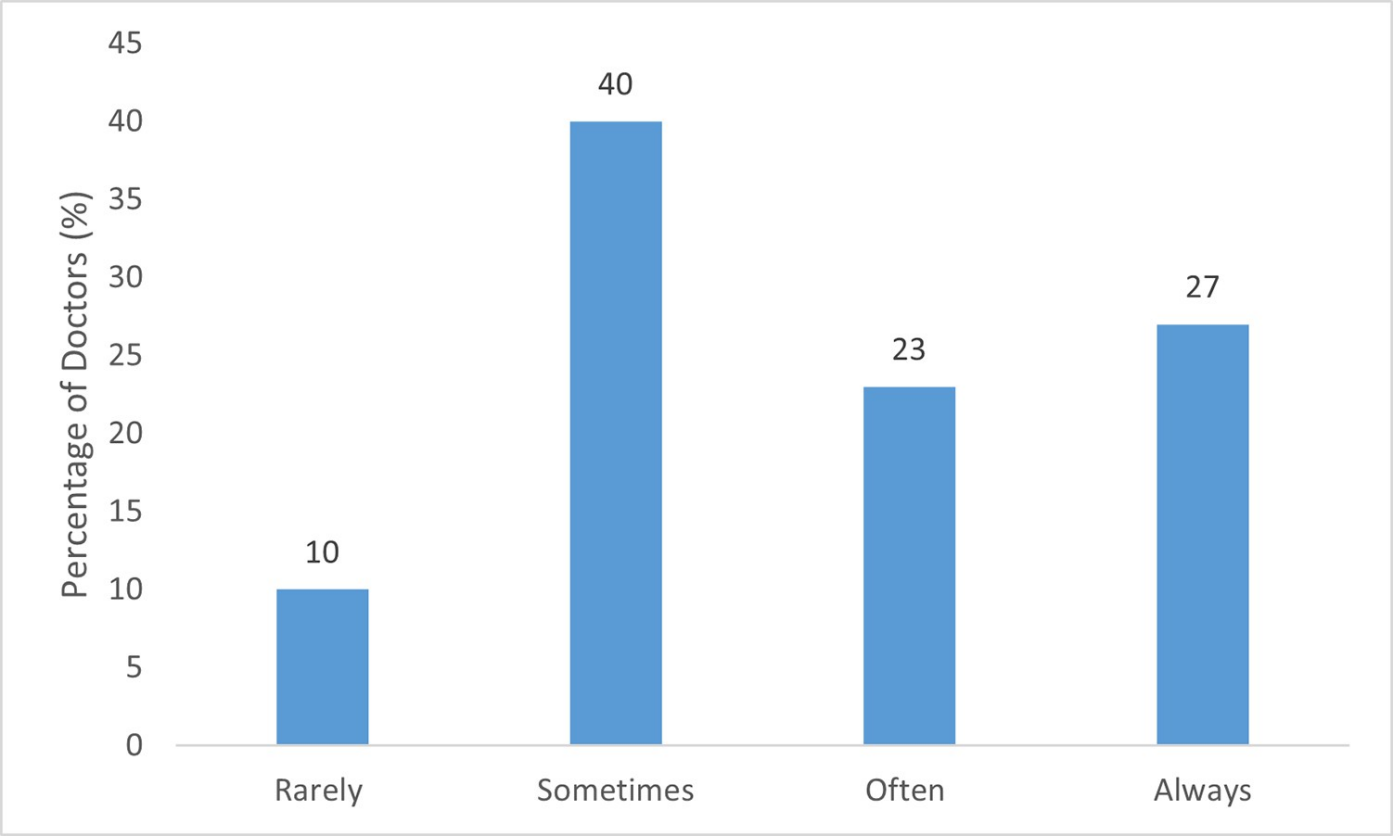
Frequency/percentage of doctors reporting return to work discussion in patient care planning.

Only 8/30 (27%) ophthalmologists reported that they always discuss return to work with their patients (Fig 1), with three (10%) reporting that they rarely take this into consideration.

**Fig 2:**
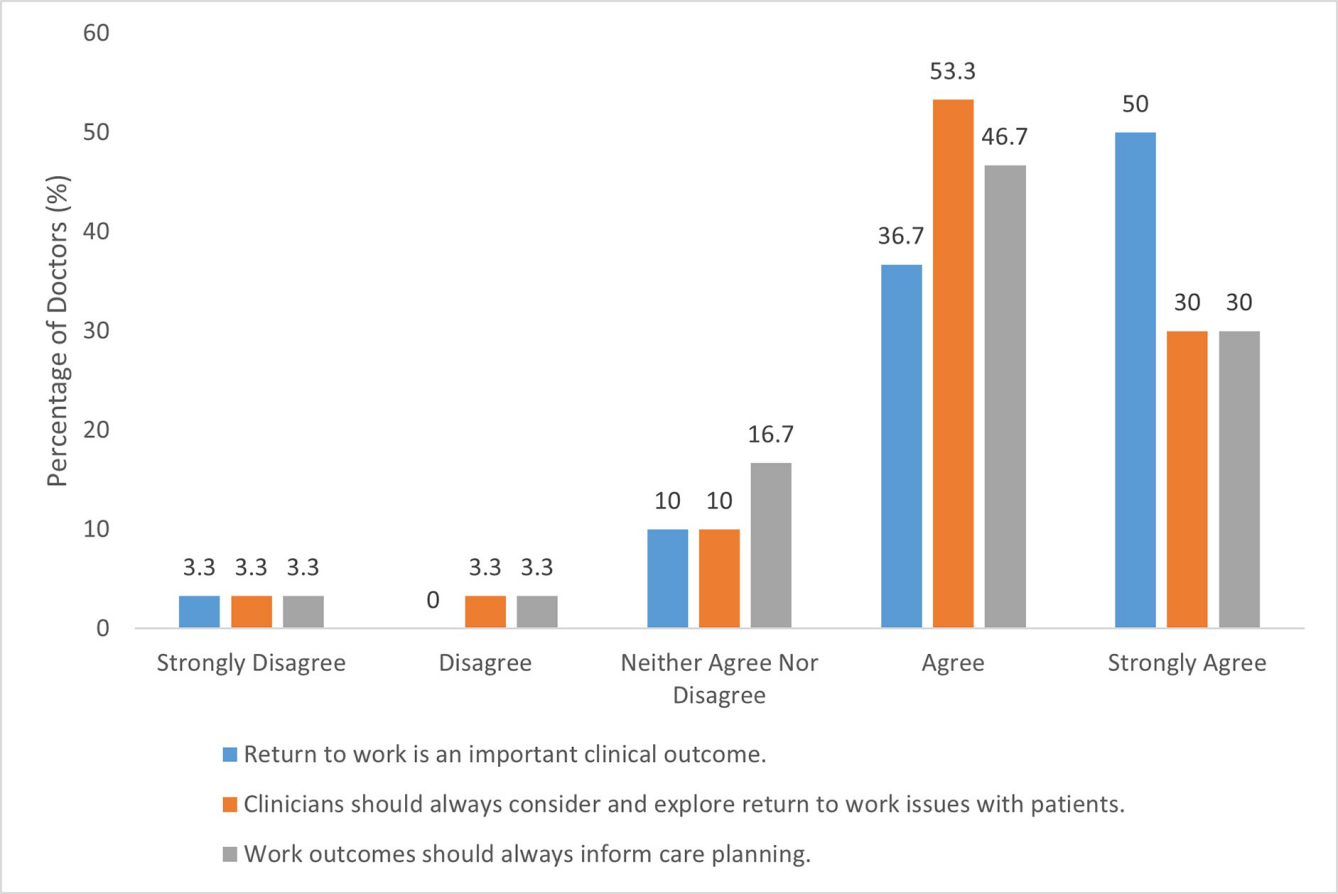
Ophthalmologists’ attitudes to return to work as a clinical outcome.

26/30 (87%) of ophthalmologists ‘agreed’ or ‘strongly agreed’ that return to work is an important clinical outcome (Fig 2) and 25/30 (83%) agreed or strongly agreed that doctors should always explore return to work with patients, with 23/30 (77%) also agreeing or strongly agreeing that work outcomes should always inform care planning (Fig 2).

**Fig 3:**
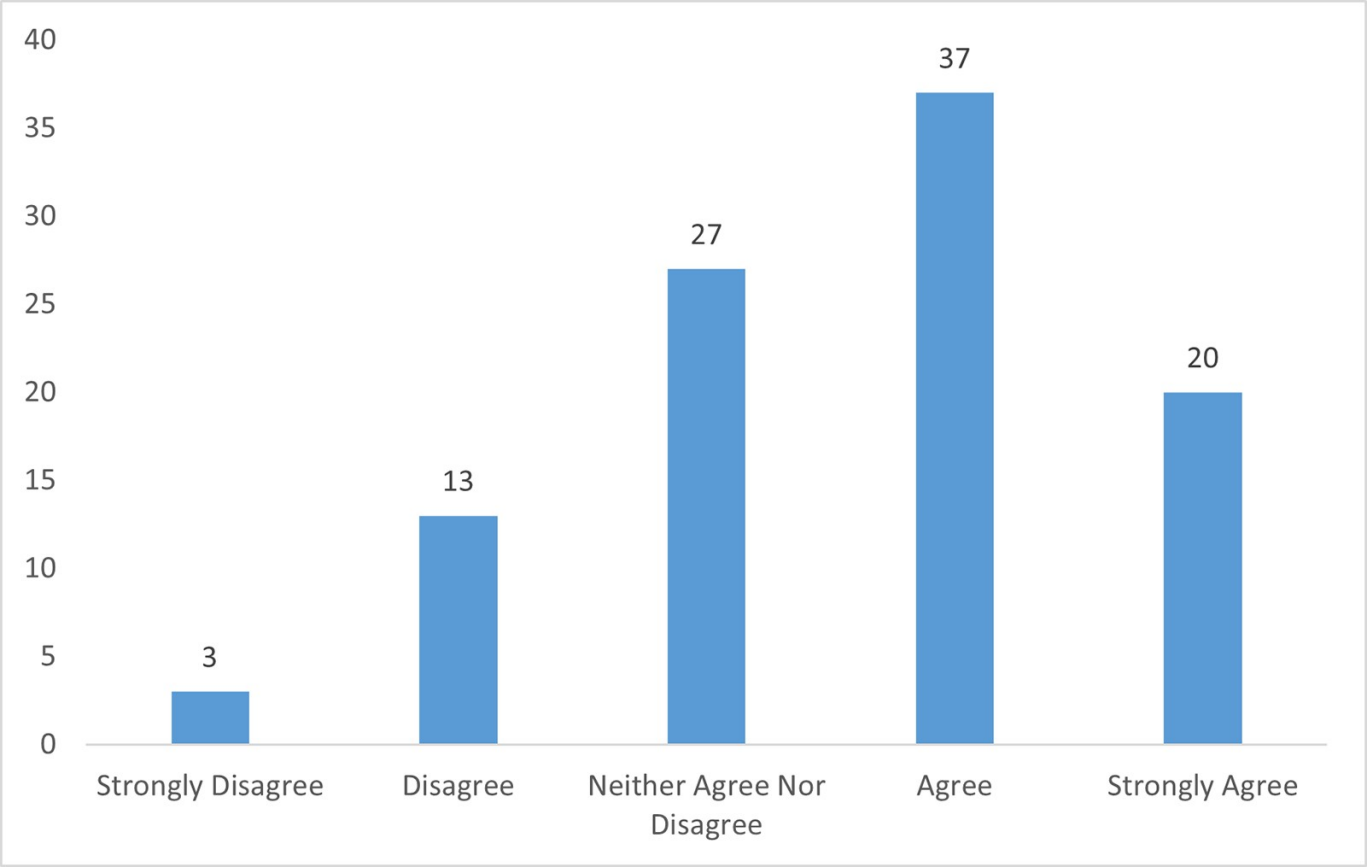
Ophthalmology doctors’ perceived competency facilitating work-focused discussions with patients as shown in percentages.

Over half (17/30;57%) agreed or strongly agreed that they were able to facilitate work-focused discussions with patients (Fig 3).

**Fig 4:**
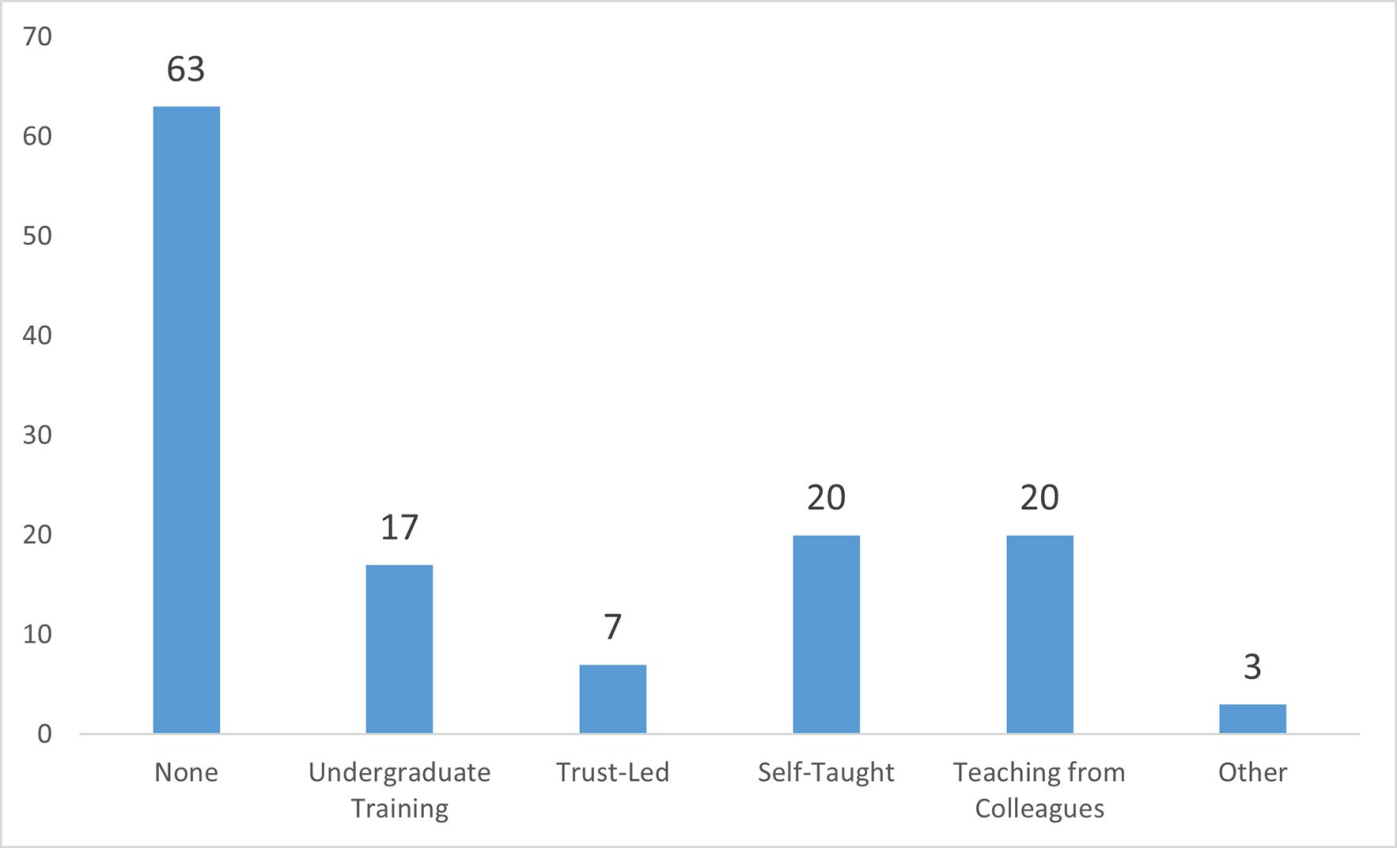
Percentage of ophthalmology doctors with previous OH training on how to discuss or assess the impact of health on work.

Nearly two-thirds (63%) of respondents did not receive any formal OH training (Fig 4), with only 2/30 (7%) reporting to have had training provided by their employer (hospital).

Over half (70%) agreed that additional OH training would be helpful for ophthalmology doctors, whereas a third (30%) of respondents were unsure or disagreed with this view. Of those who would value OH training, 21/30 (70%) recommended making it available during specialty training (Fig 4). Qualitative feedback derived from the free-text data collected highlighted that time constraints make it difficult to take a full work history and that not all eye conditions impact work functioning; in these cases, taking an OH history would not necessarily influence care-planning.

## Discussion

We found a clear lack of emphasis on work discussions during clinical practice, despite most respondents recognising the importance of this as a clinical outcome. Patients with ophthalmological conditions may be negatively affected by this lack of work-focused discussions with their treating clinician, particularly since optimal vision acuity is vital in many aspects of work. We identified a desire for OH training, particularly since many lacked confidence in facilitating work-focused discussions with patients.

An earlier study also identified a lack of, and desire for, OH training among doctors working in other specialties (namely, cardiology, gynaecology and obstetrics, oncology and orthopedics) which was also associated with feeling less competent and skilled discussing work outcomes with patients. Moreover, they found a similar discrepancy between the number of doctors from other specialties who consider it important to enquire about occupation and doing so in clinical practice [11]. Collectively these results suggest that non-OH doctors have a perference for focusing on their own specialty area during patient consultations rather than take into consideration broader factors such as the impact of health on work. This is despite work-orientated discussions during clinical consultations being recognised as important from the patient’s perspective [14].

It is important to point out that only 15% of the working-age population in the UK have access to specialist OH advice and services [16], and set within this context, while non-occupational health doctors are not required to take a detailed occupational history, there is consensus among government policy makers and OH clinicians that facilitated general work-orientated discussions by non-OH doctors can be very beneficial in supporting and enabling work participation in patients during and following periods of ill health. Specifically, by potentially reducing sickness absence duration and supporting return to work [2]. Doctor-led facilitated work-orientated discussions are a key feature of government policy reforms with regard to sickness certification in primary care and secondary care service [17, 18]. Where available, proactive specialist liaison with OH services may also guide a supported and effective return to work, particularly in safety critical roles and for more complex cases. Highlighting recovery parameters and work task restrictions within the work context could facilitate optimal return to work planning (such as appropriate work adjustments and modifications) which mitigate risk. This could not only benefit patients but also have wider social and economic benefits [1].

We recommend that future studies explore the perceived importance of work-focused discussions during consultations from the patient’s perspective which will build upon the limited existing research.

In light of the evolving evidence which has called for the enhanced provision of ‘work and health’- themed education and training for non-OH clinicians [11, 17, 19], we recommend implementing OH education during medical training for Ophthamologists to enhance clinical care.

Strengths of this study were that this was, as far as we are aware, the first study to explore the attitudes and practices of ophthamology clinicians with regard the importance of work as a clinical outcome in ophthamology clinical practice and related OH training needs. In addition we had a good response rate across all medical staffing groups within the specialty. Furthermore, our findings reflect those found from previous work in other specialties such as obstetrics and gynaecology [11]. This suggests that our collective insights may be generalisable to other surgical specialties who provide care to the working age population. Notwithstanding, we also acknowledge that the main limitation with this study was the non-random and self-selected sample population who took part coupled with the potential impact of recall bias.

## Conclusion

To date, there remains a lack of research exploring non-occupational health doctors’ attitudes towards discussing occupation and return to work with patients.

In this present work, we found that although the majority of ophthalmology doctors placed great importance on return to work for patients, they do not routinely take into consideration a patient’s occupation or job tasks as part of their clinical consultations and when care planning. This study highlighted the extent to which focus on return to work is lacking among ophthalmologists, and the need for OH training in the specialty to enhance care planning.

## Supporting information

S1 Data (XLSX)
